# Semiautomated and Automated Quantitative Analysis of Corneal Sub-Basal Nerves in Patients With DED With Ocular Pain Using IVCM

**DOI:** 10.3389/fmed.2022.831307

**Published:** 2022-02-11

**Authors:** Yu Zhang, Yaying Wu, Wenbo Li, Xiaodan Huang

**Affiliations:** Eye Center, School of Medicine, The Second Affiliated Hospital of Zhejiang University, Hangzhou, China

**Keywords:** dry eye disease, ocular pain, *in vivo* confocal microscopy, corneal subbasal nerve plexus, NeuronJ, ACCMetrics

## Abstract

**Purpose:**

Investigate the correlation and agreement between the results of semiautomated and fully automated quantitative analysis of the corneal sub-basal nerve plexus (SNP) in patients with dry eye disease (DED) with ocular pain using *in vivo* confocal microscopy (IVCM).

**Method:**

A total of 50 voluntary participants were enrolled in this study, i.e., 25 DED patients with ocular pain and 25 healthy controls. Each patient underwent an evaluation of ocular symptoms that utilized: the Ocular Surface Disease Index (OSDI), the Ocular Pain Assessment Survey (OPAS), the tear film breakup time (TBUT) test, the Schirmer test, corneal staining, and IVCM. Five SNP images of the cornea of each eye were selected and analyzed using a semiautomated analysis software (NeuronJ) and a fully automated method (ACCMetrics) to quantify corneal nerve fiber density (CNFD), corneal nerve branch density (CNBD), and corneal nerve fiber length (CNFL).

**Results:**

The intraclass correlation coefficient (ICC) of the CNFD (0.460 [0.382–0.532], *p* < 0.001), CNBD (0.608 [0.545–0.665], *p* < 0.001), and CNFL (0.851 [0.822–0.875], *p* < 0.001) represents the repeatability and consistency of measurements by the NeuronJ and ACCMetrics software. The CNFL values (r = 0.881, *p* < 0.001) obtained using the two methods have extremely high correlation, and similarly, the CNFD values (r = 0.669, *p* < 0.001) and CNBD values (r = 0.703, *p* < 0.001) are highly correlated. The CNFL had the biggest area under the curve (AUC; 0.747 [0.700–0.793], *p* < 0.001) when using ACCMetrics. In DED patients with ocular pain, the mean CNFD values for semiautomated and fully automated quantization were 23.5 ± 8.1 and 23.8 ± 8.6 n/mm^2^; the mean CNBD values were 46.0 ± 21.3, 35.7 ± 23.3 n/mm^2^; and the mean CNFL values were 19.3 ± 4.3 and 15.2 ± 3.8 mm/mm^2^, which were significantly lower than healthy subjects (*p* < 0.001).

**Conclusion:**

There is a significant correlation between the measurements obtained via ACCMetrics and NeuronJ, especially for CNFL, which can be considered as the primary indicator in the diagnosis of DED with ocular pain. The SNP of the disease was significantly lower than that of healthy subjects.

## Introduction

Dry eye disease (DED) is one of the most often reported ocular diseases, with a global incidence of 5–50% by symptoms (1–3). Ocular surface inflammation and injury, tear film instability, hyperosmolarity, and neurosensory abnormalities are all factors that contribute to DED, as defined by the International Dry Eye Workshop in 2017 (4). Along with abnormalities of the tear film and ocular surface, the revised DED criteria include neurosensory dysfunction as a contributing component. Ocular pain, such as burning, aching, and itching, is a frequent symptom of moderate-to-severe DED. It is commonly accepted that DED combined with ocular pain alters the structure and function of the ocular surface's sensory nerves, resulting in the appearance of unpleasant sensations ranging from mild discomfort and dryness to scorching pain (5).

With an abundant supply of nerve fibers, the cornea is one of the most highly innervated tissues in the body. The human cornea is filled with sensory nerve fibers originating from the trigeminal nerve's ophthalmic branch and sympathetic and parasympathetic nerve fibers. Nerve fibers in the human cornea flow radially from the periphery of the anterior part of the stroma to its center. These nerve fibers penetrate the Bowman's layer and then branch vertically and horizontally between the epithelium of the Bowman's layer and the basal epithelium to create a network of nerve fibers known as the sub-basal nerve plexus (SNP) (6). Numerous scientific investigations have been conducted on the corneal nerve's structural and functional anomalies in the setting of a range of ocular and systemic illnesses (7).

Corneal *in vivo* confocal microscopy (IVCM) is a noninvasive technique for obtaining high-resolution images of the SNP at the cellular level with image quality equivalent to that obtained using histochemical techniques. Oliveira-Soto and Efron were the first to utilize IVCM to describe corneal nerves (8). Previous research has revealed variations in the morphology of the corneal nerves in patients having DED, such as considerably decreased nerve density and relatively high reflectivity, beading, and tortuosity. Shetty et al. observed a substantial reduction in SNP characteristics (corneal nerve fiber length [CNFL], fiber density, fiber width, total branch density, nerve branch density, and fiber area) in DED with ocular pain patients (9). Yavuz-Saricay et al. discovered a significant decrease in main and branch nerve densities, an increase in dendritic cell density, and the existence of microneuromas in DED patients with ocular pain (10).

Notwithstanding the growing use of IVCM in DED clinical practice, there have only been a few pertinent advances in available software tools that facilitate automated corneal nerve analysis. To date, the majority of IVCM image analyses of corneal nerves have been performed manually or semiautomatically, which has a lot of drawbacks, such as being time-consuming, subjective, prone to observer bias, and having low repeatability and consistency (6). According to the latest the dry eye workShop II (DEWS II) of the tear film and ocular surface society (TFOS) Pain and Sensation Subcommittee Report, automating quantitative IVCM assessments would significantly improve research technique and interpretation of the results (5).

At the present, quantitative applications for evaluating corneal nerves range from fully manual (CCMetrics, University of Manchester, Manchester, UK) to semiautomated (NeuronJ, with ImageJ plugin, National Institutes of Health, Bethesda, MA, USA) to fully automated (ACCMetrics, University of Manchester, Manchester, UK), all of which generate varying degrees of quantitative nerve evaluation (11). Due to the laborious and time-consuming nature of applying fully automated CCMetrics software, the fully automated CCMetrics approach has been replaced with the semiautomated NeuronJ technique (12). The NeuronJ software traces the structure of the nerve semiautomatically with a smooth line, providing total length measurements. However, the operator's ability to discern the nerve's beginning and end impacts the accuracy of the measurements (13). ACCMetrics is a fully automated image analysis program that reduces the time required to evaluate an image to 15 s per image. ACCMetrics evaluates neural morphometric characteristics, such as corneal nerve fiber density (CNFD), CNFL, and corneal nerve branch density (CNBD), and nerve fiber total branch density, nerve fiber area, and nerve fiber width. As a result, it is particularly advantageous for major studies analyzing a massive amount of IVCM images (14).

The objective of this study is to evaluate the diagnostic performance of ACCMetrics, a fully automated software, with that of NeuronJ, a semiautomated program, in distinguishing patients with DED with ocular pain from healthy controls via IVCM morphometric analysis of the corneal SNP. The correlations between DED with ocular pain and several characteristics of the corneal SNP, such as the CNFD, CNBD, and CNFL, were also investigated to identify which was the more significant diagnostic parameter.

## Materials and Methods

### Subjects

We conducted this prospective observational study in 2019 and 2020 at the outpatient clinics of the Second Affiliated Hospital, Zhejiang University School of Medicine, in adherence to the precepts of the Declaration of Helsinki. The study was authorized by the Ethics Committee of the Second Affiliated Hospital at Zhejiang University School of Medicine (2019-283). All participants freely signed an informed consent form. A total of 50 participants comprised DED patients (n = 25) and healthy controls (*n* = 25).

An OSDI score of ≥13 points, a Schirmer I test score of <10 mm/5 min, and a tear film breakup time (TBUT) of <10 s, associated with Ocular Pain Assessment Survey (OPAS) scores of 10–27, were used as diagnostic criteria for DED with ocular pain.

An OSDI score of <7 points, a Schirmer I test score of ≥10 mm/5 min, or a TBUT score of ≥10 s, associated with OPAS scores of 0–9, is considered healthy.

Individuals who met the following criteria were all excluded from the study: (1) inability or unwillingness to sign an informed consent form, (2) a history of eye surgery, (3) a history of wearing contact lenses during the previous 30 days, (4) a history of eye diseases other than DED, (5) a history of using any eye medication other than eye lubricant during the previous 3 months, and (6) severe systemic disease.

### *In vivo* Confocal Microscopy

In this study, all participants underwent an IVCM examination. The remote center of motion (RCM) module of the *in vivo* confocal microscope (HRT-3, Heidelberg Engineering GmbH, Heidelberg, Germany) was used to observe the center of the corneal epithelium. The IVCM has a helium-neon diode laser with a wavelength of 670 nm, a × 60 objective lens (Olympus, Tokyo, Japan), a numerical aperture of 0.9, and a working distance of 0.0–3.0 mm relative to the flat cap (Tomo-Cap, Heidelberg Engineering GmbH, Germany). The images were two-dimensional, with a field size of 400 × 400 μm and a resolution of 384 × 384 pixels. An expert technician performed all operations. Before beginning the examination, the technician placed a drop of carbomer gel (Bausch & Lomb, Germany) on the microscope lens and then covered it with a disposable corneal contact cap. Next, a drop of topical anesthetic was applied to the eye (Proparacaine Hydrochloride Eye Drops, Alcon, United States), and the participant was instructed to place their chin and forehead in a bracket. Participants were instructed to look at a blip of light directly from the machine in front of them. The lens was zeroed out before being pushed toward the cornea of the participant's eye. The SNP of the cornea was then targeted at a depth of 50–80 μm. The participants were then instructed to move their center of attention across their visual field to capture images of the corneal nerves from the central, upper, lower, nasal, and temporal orientations. A minimum of five clean, wrinkle-free, nonoverlapping, and representative photos were taken in each direction. The five most representative photos for each eye were selected and examined using NeuronJ and ACCMetrics. NeuronJ is a Java-based image analysis software package that includes a nerve-tracing plugin module. ACCMetrics is a software application for autonomous image analysis and processing developed by the University of Manchester in Manchester, United Kingdom. All images were manually assessed using NeuronJ by two experienced *blind observers* (YZ and WL). CNFD, CNBD, and CNFL were chosen as measurement indicators.

### Ocular Surface Disease Index (OSDI)

The OSDI consists of six questions on visual disturbance and visual function. Each response is graded on a 5-point scale, with the total OSDI score ranging from 0 (no symptoms) to 100 (maximum symptoms). Patients with a score >13 are diagnosed with symptomatic DED.

### Ocular Pain Assessment Survey

We assessed average eye pain severity and frequency, aggravating causes, related factors, and symptomatic alleviation over the preceding 2 weeks using a 32-question ocular pain questionnaire designed by Yureeda Qazi using numerical rating scales (15).

### Tear Film Breakup Time

The TBUT test assesses the amount of time between a full blink and the appearance of the first tear film break.

### Corneal Fluorescein Staining

Corneal fluorescein staining was conducted after the TBUT test. The Oxford Scale was used to assess corneal stains under a yellow filter. Positive scores were defined as those that exceeded zero points.

### Statistics

All statistical analyses, excluding Bland-Altman plots, were conducted using version 26 of the IBM Statistical Package for the Social Sciences (SPSS) software package for Windows (IBM, Chicago, IL, USA). Bland-Altman plots were created via an online website: https://spssau.com. IVCM and SPSS were used to construct the figures used in this study. The descriptive statistics are summarized as mean ± SD. A Kolmogorov-Smirnov test was used to assess the normality of continuous variables, and the Bland-Altman plots were employed to determine the interobserver agreement between the measurements. The repeatability and reproducibility values were calculated in terms of mean bias and 95% limits of agreement (LoA). The intraclass correlation coefficient (ICC) was determined as an index of repeatability and reproducibility between measurements. Paired *t*-tests were used to assess the differences between the two sets of measurements being compared. The receiver operating characteristic (ROC) curve, area under the ROC curve (AUC), cutoff point, sensitivity, and specificity were also calculated. Correlation between the results obtained using NeuronJ and ACCMetrics was explored using logarithmic Pearson's correlation coefficient (r). The analysis was double-sided, and a *p* < 0.05 was considered statistically significant.

## Results

### Demographics and Medical Status

A total of 50 voluntary participants were enrolled in this study, comprising 25 patients with DED with ocular pain (7 men and 18 women, mean age: 43.8 ± 14.7 years) and 25 healthy controls (6 men and 19 women, mean age: 44.2 ± 11.0 years). The clinical test results of patients with DED with ocular pain were as follows: average pain time of 1.89 ± 1.1 years, average OSDI score of 62.73 ± 20.73, average OPAS score of 12.84 ± 5.77, average TBUT of 2.24 ± 1.27 s, average Schirmer test score of 8.18 ± 7.36 mm/5', and average corneal staining score of 1.89 ± 1.59. These indicators were within normal limits in the healthy controls.

### Interobserver Agreement for NeuronJ and ACCMetrics Measurements

All the images were measured by two blind observers (YZ and WL), semiautomatically using NeuronJ and automatically using ACCMetrics.

Table 1 presents a summary of the ICC for interobserver measures obtained using NeuronJ and ACCMetrics to show the amount of agreement between the two observers. As can be seen in Table 1, all three parameters had an excellent interobserver agreement. With an ICC value of 0.933 (95% CI: 0.914–0.948, *p* < 0.001), CNFD had the most agreement, followed by CNBD, with an ICC value of 0.921 (95% CI: 0.898–0.938, *p* < 0.001), and CNFL, with an ICC value of 0.918 (95% CI: 0.895–0.937, *p* < 0.001).

**Table 1:** The intraclass correlation coefficient (ICC) for measurements of NeuronJ and ACCMetrics.

**ICC**	**Neuron J**		**ACCMetrics**	
	**Interobserver**	***p*** **value**	**Comparison with**	***p*** **value**
			NeuronJ^*^	
CNFD	0.933 (0.914–0.948)	<0.001	0.460 (0.382–0.532)	<0.001
CNBD	0.921 (0.898–0.938)	<0.001	0.608 (0.545–0.665)	<0.001
CNFL	0.918 (0.895–0.937)	<0.001	0.851 (0.822–0.875)	<0.001

* *Comparisons between ACCMetrics and mean measurements from two observers*.

*Results are expressed as mean ± SD, statistically significant difference using ANOVA. In brackets are the 95% CIs*.

**Figure 1:**
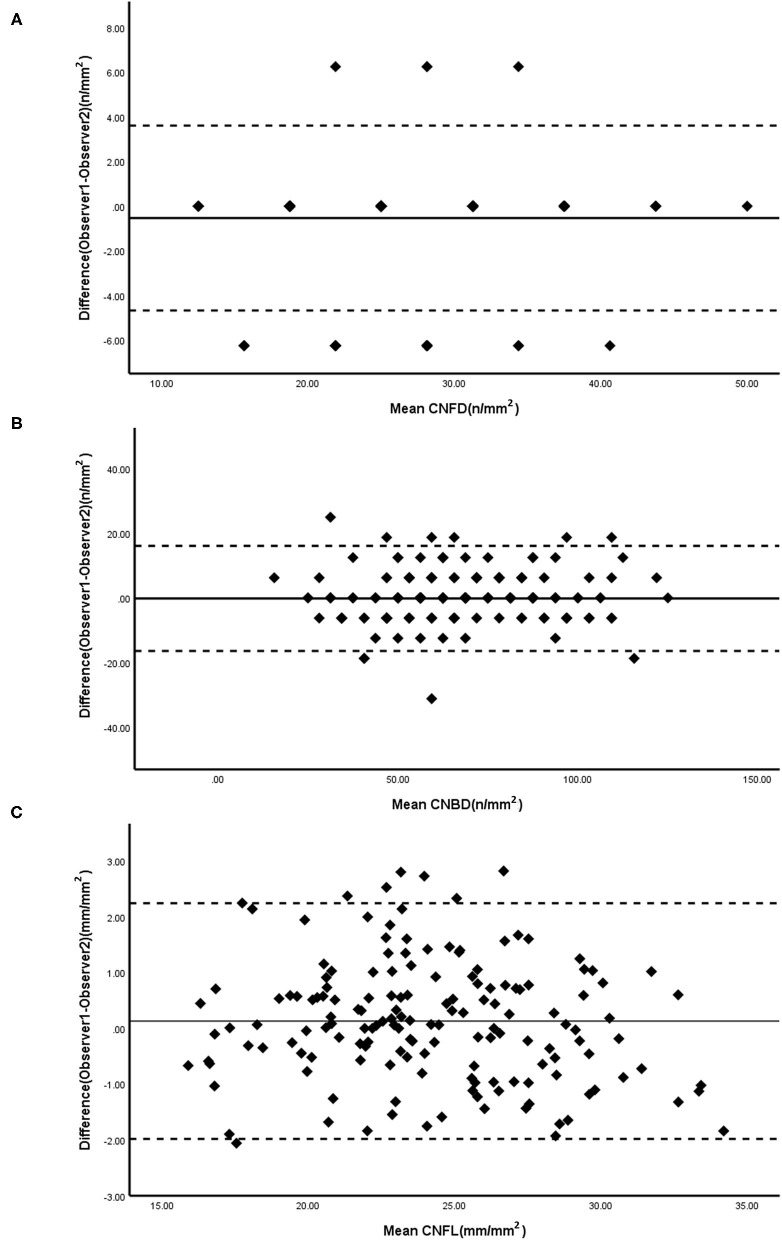
Bland-Altman plots for CNFD **(A)**, CNBD **(B)**, and CNFL **(C)** were measured by two masked observers. Solid line, mean difference; Dashed lines, 95% CI. CNFD, corneal nerve fiber density; CNBD, corneal nerve branch density; CNFL, corneal nerve fiber length.

Figure 1 presents Bland-Altman plots of differences vs. the averages of the CNFD, CNBD, and CNFL in images of the healthy controls obtained by the blind observers, YZ and WL. The solid lines indicate the average difference, while the dashed lines represent the 95% LoA. The mean difference (±SD) between the two observers was as follows: CNFD, −0.533 ± 2.113; CNBD, 0.147 ± 5.202; and CNFL, 0.130 ± 1.078. The coefficients of repeatability (CoR) for CNFD, CNBD, and CNFL were 4.262, 10.176, and 2.121, respectively.

### Differences in CNFD, CNBD, and CNFL Between the Semiautomated and Automated Methods

A total of 500 images obtained from the entire cohort were analyzed using NeuronJ and ACCMetrics. The data on CNFD, CNBD, and CNFL are presented in Table 2. The mean CNFDs quantified via NeuronJ and ACCMetrics were 25.0 ± 7.6 and 27.4 ± 9.4 n/mm^2^, respectively (paired-samples *t*-test, t = −5.619, *p* < 0.001). The mean CNBDs quantified via NeuronJ and ACCMetrics were 57.1 ± 23.3 and 41.1 ± 24.2 n/mm^2^, respectively (paired-samples *t*-test, t = 15.740, *p* < 0.001), and the mean CNFL quantified via NeuronJ and ACCMetrics were 21.6 ± 4.7 and 17.0 ± 4.0 mm/mm^2^, respectively (paired-samples *t*-test, t = 40.385, *p* < 0.001). As can be seen in Table 2, there is a substantial difference between the results obtained *via* the two strategies. The CNFD calculated by ACCMetrics was significantly higher than that assessed via NeuronJ, while the CNBD and CNFL estimated by ACCMetrics were clearly lower than those calculated via NeuronJ.

**Table 2:** Mean ± SD of parameters of the entire cohort measured by NeuronJ and ACCMetrics.

**TOTAL**	**NeuronJ**	**ACCMetrics**	**t Value**	***p*** **Value**
CNFD (n/mm^2^)	25.0 ± 7.6	27.4 ± 9.4	−5.619	<0.001
CNBD (n/mm^2^)	57.1 ± 23.3	41.1 ± 24.2	15.740	<0.001
CNFL (mm/mm^2^)	21.6 ± 4.7	17.0 ± 4.0	40.385	<0.001

*Results are expressed as mean ± SD*.

**Figure 2:**
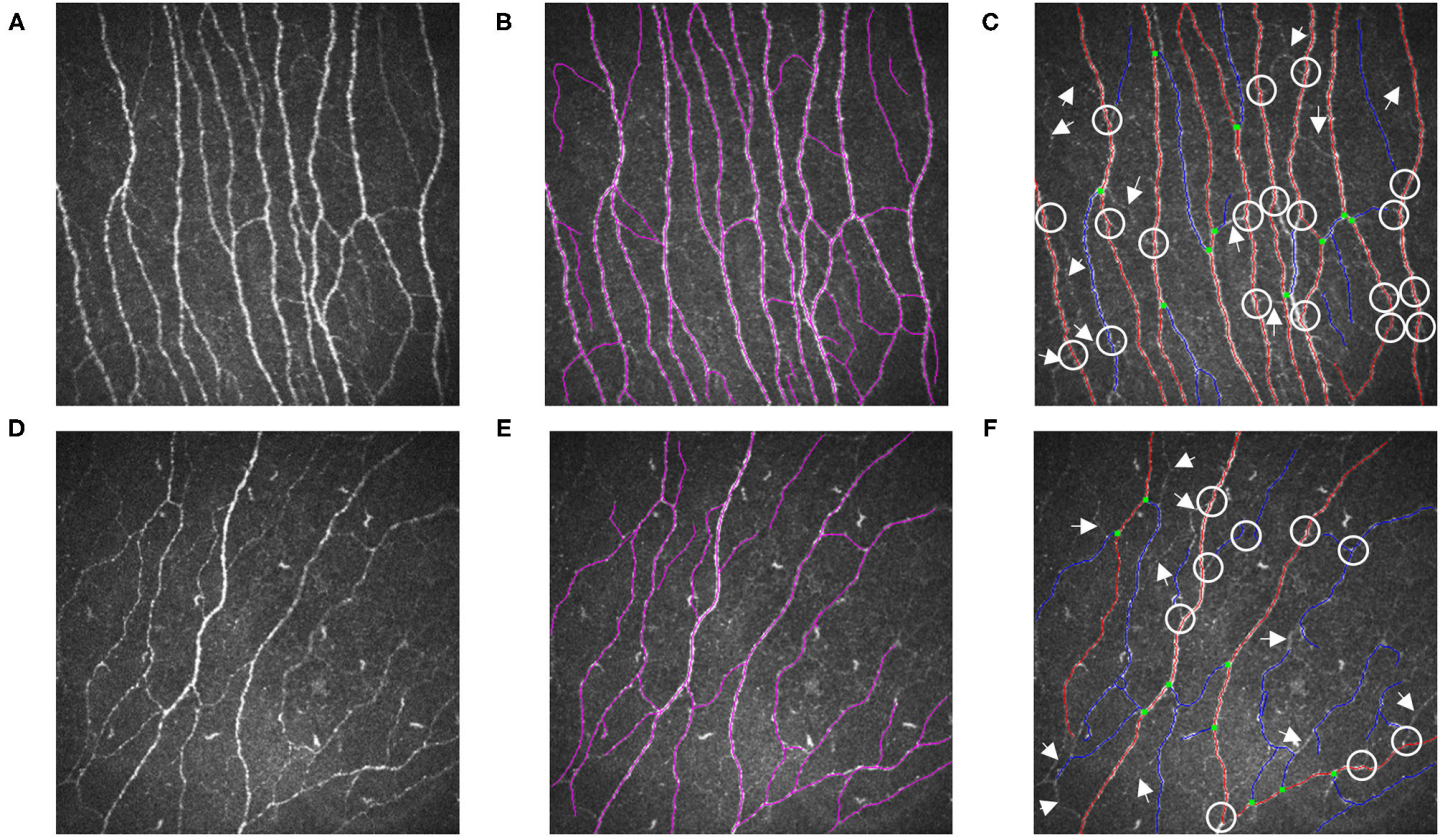
Illustrations are examples of SNP measurements. **(A)** A representative raw IVCM image from a healthy control and **(D)** a patient of DED with ocular pain. **(B,E)** were measured by NeuronJ; **(C,F)** were automatically analyzed by ACCMetrics. Nerve fibers which failed to be identified were marked as arrows. Nerve branches that were unable to be detected were marked as circles. DED, dry eye disease; SNP, sub-basal nerve plexus; IVCM, *in vivo* confocal microscopy.

As illustrated in Figure 2, the nerve fibers traced in NeuronJ are colored purple, and NeuronJ is capable of calculating the sum of the fiber lengths (CNFL). However, the CNFD and CNBD values need to be manually recorded by the observers. In the images analyzed using ACCMetrics, CNFD values were traced in red, CNBD values were denoted as green dots, and CNFL is the sum of all nerve tracings, i.e., red-colored tracings and blue-colored tracings. To a certain extent, some of the CNBD and CNFL values were not detected and are indicated by arrows and circles in the images.

### Agreement in CNFD, CNBD, and CNFL Between NeuronJ and ACCMetrics

Table 1 presents a summary of the ICC for the two methods: NeuronJ and ACCMetrics. An ICC value of 0.460 (95% CI: 0.382–0.532, *p* < 0.001) was the lowest value for the CNFD. The CNBD has an ICC value of 0.608 (95% CI: 0.545–0.665, *p* < 0.001), while the CNFL has an ICC value of 0.851 (95% CI: 0.822–0.875, *p* < 0.001). We can speculate that the CNFL has strong agreement and repeatability.

**Figure 3:**
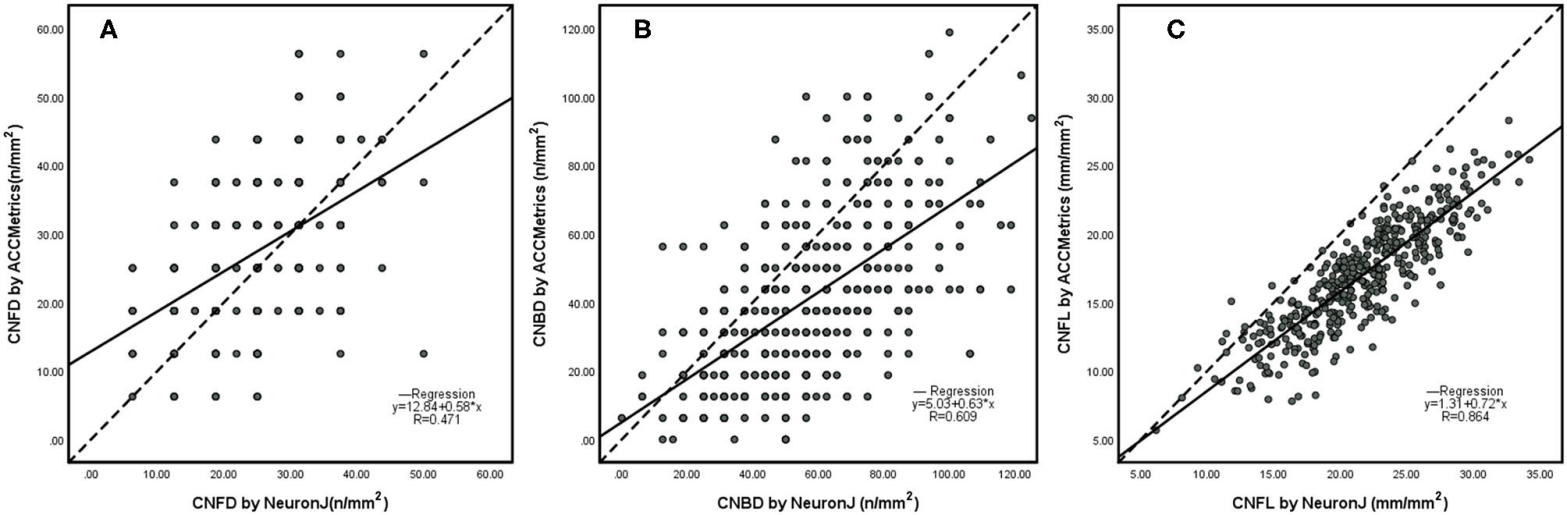
Scatterplots of CNFD **(A)**, CNBD **(B)**, and CNFL **(C)** using NeuronJ vs. ACCmetrics on the whole images (*n* = 500). CNFD, corneal nerve fiber density; CNBD, corneal nerve branch density; CNFL, corneal nerve fiber length.

The scatterplots (Figure 3) of the CNFD, CNBD, and CNFL showed correlations between NeuronJ and ACCMetrics. The CNFD values (r = 0.471, *p* < 0.001) are moderately related, the CNBD values (r = 0.609, *p* < 0.001) are strongly related, and the CNFL values (r = 0.864, *p* < 0.001) are highly related, based on manual and automated analysis.

**Figure 4:**
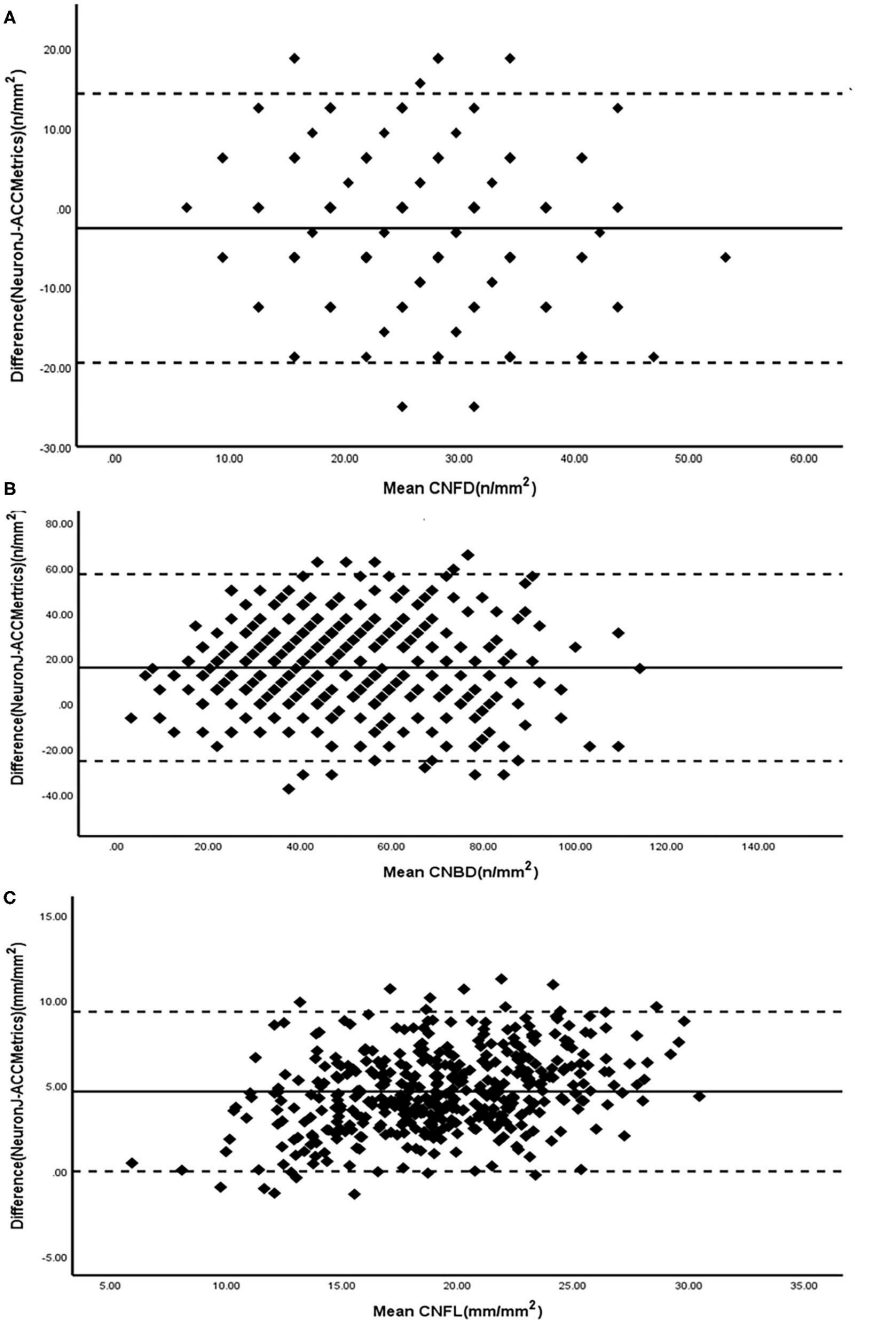
Bland-Altman plots for CNFD **(A)**, CNBD **(B)**, and CNFL **(C)** measured by NeuronJ and ACCMetrics. Solid lines, mean difference; Dashed lines, 95% CI. CNFD, corneal nerve fiber density; CNBD, corneal nerve branch density; CNFL, corneal nerve fiber length.

The Bland-Altman plots (Figure 4) for the CNFD, CNBD, and CNFL present the agreements between the results obtained via NeuronJ and ACCMetrics. The results show differences in CNFD (−2.4 ± 8.9), CNBD (16.0 ± 21.0), and CNFL (4.7 ± 2.4); the 95% LoA of CNFD (−19.903 – 15.058), CNBD (−25.207 – 57.193), and CNFL (−0.013 – 9.348); and the repeatability coefficient (RC) of CNFD (18.094), CNBD (51.730), and CNFL (10.273). The majority of the scatter plots fall within the 95% CI, indicating that the two detection methods are consistent.

**Figure 5:**
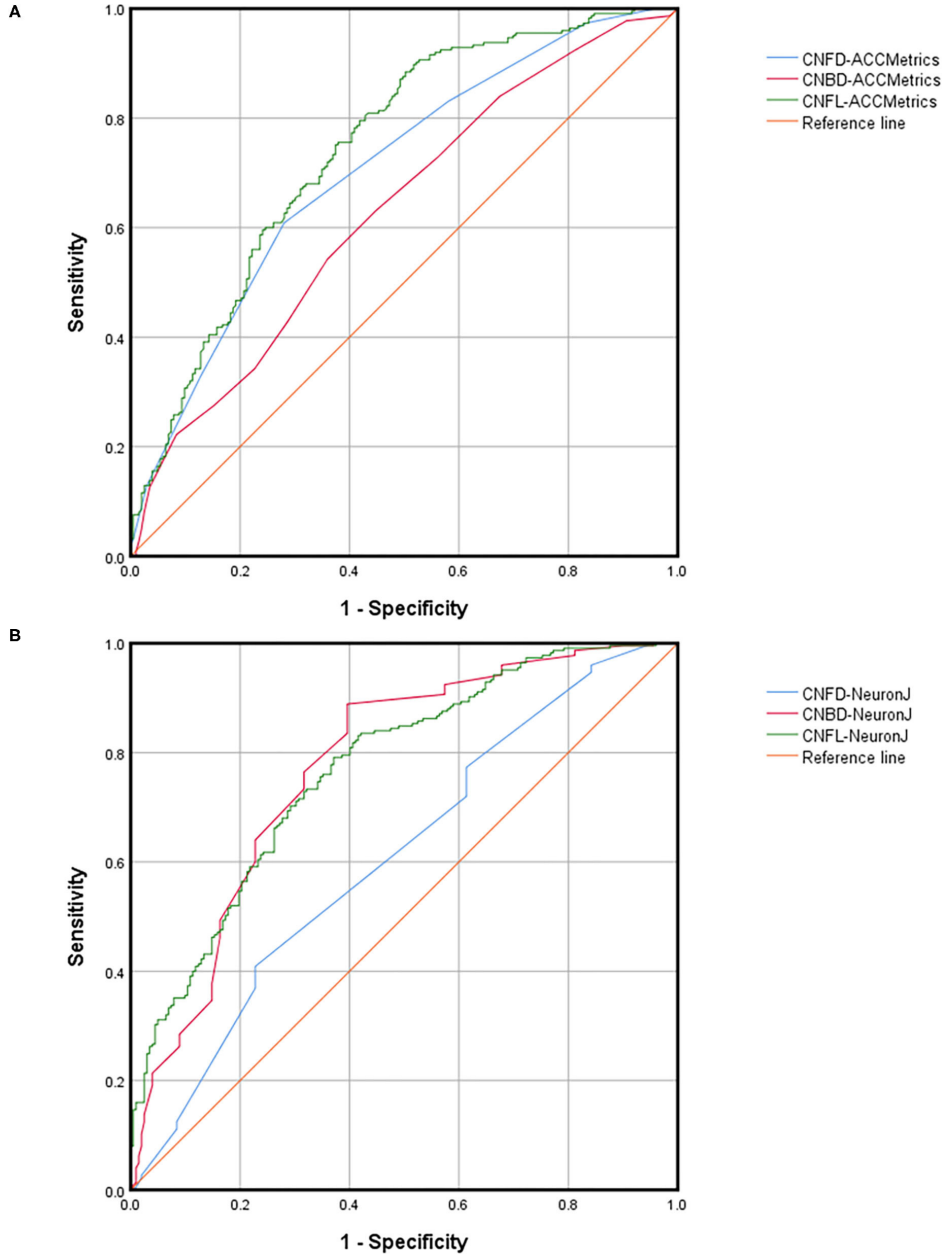
ROC parameters of CNFD, CNBD, and CNFL dividing by DED with ocular pain using ACCMetrics **(A)** and NeuronJ **(B)**. DED, dry eye disease; CNFD, corneal nerve fiber density; CNBD, corneal nerve branch density; CNFL, corneal nerve fiber length.

The ROC curves and relevant analysis convincingly demonstrate the ability of each parameter to diagnose DED with ocular pain. Table 3 presents a summary of the AUC, cutoff, sensitivity, and specificity values, while Figure 5 presents the ROC curves. Using ACCMetrics, the CNFL most effectively distinguishes patients with DED with ocular pain from healthy controls (AUC = 0.747 [0.700–0.793], cutoff = 0.384, sensitivity = 0.884, specificity = 0.500, *p* < 0.001). Using NeuronJ, the CNBD had the highest AUC of 0.773 (0.728–0.818), followed by the CNFL (0.769 [0.725–0.813]) and CNFD (0.608 [0.555–0.662]).

**Table 3:** AUC indicators for the discrimination of DED with ocular pain.

**Parameter**	**AUC & 95%CI**	**Cutoff**	**Sensitivity**	**Specificity**	***p*** **Value**
ACCMetrics –CNFD	0.705 (0.656–0.754)	0.328	0.609	0.719	<0.001
ACCMetrics –CNBD	0.629 (0.577–0.682)	0.183	0.631	0.552	<0.001
ACCMetrics –CNFL	0.747 (0.700–0.793)	0.384	0.884	0.500	<0.001
NeuronJ–CNFD	0.608 (0.555–0.662)	0.177	0.409	0.768	<0.001
NeuronJ–CNBD	0.773 (0.728–0.818)	0.490	0.889	0.601	<0.001
NeuronJ–CNFL	0.769 (0.725–0.813)	0.420	0.791	0.629	<0.001

*AUC, area under the ROC curve; DED, dry eye disease*.

### Significantly Lower CNFD, CNBD, and CNFL in Patients With DED With Ocular Pain Than in Healthy Controls *via* Both NeuronJ and ACCMetrics

To investigate the correlation between SNP and patients with DED with ocular pain, we compared the mean differences between the results obtained using NeuronJ and ACCMetrics for healthy controls and DED with ocular pain patients. As illustrated in Table 4, the patients with DED with ocular pain had significantly fewer SNP than healthy controls. When using NeuronJ, the values for the CNFD, CNBD, and CNFL were 26.5 ± 6.9 vs. 23.5 ± 8.1 n/mm^2^, *p* < 0.001; 67.0 ± 20.4 vs. 46.0 ± 21.3 n/mm^2^, *p* < 0.001; and 23.7 ± 4.1 vs. 19.3 ± 4.3 mm/mm^2^, *p* < 0.001, respectively. When ACCMetrics is utilized, the values for CNFD, CNBD, and CNFL were 30.7 ± 8.9 vs. 23.8 ± 8.6 n/mm^2^, *p* < 0.001; 46.0 ± 24.0 vs. 35.7 ± 23.3 n/mm^2^, *p* < 0.001; and 18.6 ± 3.4 vs. 15.2 ± 3.8 mm/mm^2^, *p* < 0.001, respectively.

**Table 4:** Mean ± SD of parameters measured by NeuronJ and ACCMetrics.

		**Control**	**DED with ocular pain**	**Mean difference & 95%CI**	***t*** **Test**	
					***t*** **Value**	***P*** **Value**
CNFD (n/mm^2^)	NeuronJ	26.5 ± 6.9	23.5 ± 8.1	3.0 (1.6–4.4)	4.1	<0.001
	ACCMetrics	30.7 ± 8.9	23.8 ± 8.6	6.9 (5.2–8.6)	8.1	<0.001
CNBD (n/mm^2^)	NeuronJ	67.0 ± 20.4	46.0 ± 21.3	20.9 (16.9–24.9)	10.4	<0.001
	ACCMetrics	46.0 ± 24.0	35.7 ± 23.3	10.3 (5.8–14.8)	4.5	<0.001
CNFL (mm/mm^2^)	NeuronJ	23.7 ± 4.1	19.3 ± 4.3	4.5 (3.7–5.3)	11.0	<0.001
	ACCMetrics	18.6 ± 3.4	15.2 ± 3.8	3.4 (2.7–4.1)	9.7	<0.001

*Results are expressed as mean ± SD. DED, dry eye disease*.

## Discussion

In this study, we compared two distinct assessment techniques for IVCM images and the difference in SNP between DED with ocular pain patients and healthy subjects. We used ACCMetrics, a fully automated measurement software, and NeuronJ, a semiautomated method, to identify SNP and detect ocular pain symptoms associated with DED with ocular pain.

Previous research studies have shown that patients with DED with ocular pain have fewer SNP than healthy controls and that ACCMetrics can partially replace the manual method. The semiautomated measurements were particularly well correlated with the measurements obtained via the manual methods for the same image (16). According to a study by Giannaccare et al., the ACCMetrics software can detect alternations in SNP and distinguish DED patients (6). In patients with DED with ocular pain, Kheirkhah et al. discovered a significantly low number and density of sub-basal nerves (17). However, research studies on the relationship between SNP and ocular pain symptoms in DED with ocular pain patients, and the corresponding measurement indicators, were hard to obtain. Simultaneously, it is necessary to demonstrate the specific efficacy of fully automated software over the traditional method.

The ACCMetrics software can partially replace semiautomated methods (18). The traditional semiautomated method has a high ICC and CoR between observers for the CNFD, CNBD, and CNFL (Table 1 and Figure 1). In Table 1, it can also be seen that the CNFL (ICC = 0.851, *p* < 0.001) has the highest ICC between ACCMetrics and Neuron J, whereas the CNBD (ICC = 0.608, *p* < 0.001) has a medium ICC. Between the two methods, the CNFL has the highest correlation (r = 0.864, *p* < 0.001), followed by the CNBD (r = 0.609, *p* < 0.001). The difference in the CNFL (4.7 ± 2.4) is apparent on the Bland-Altman plot, and the majority of the scatter plots are distributed within the 95% CI. Based on the foregoing, we propose the CNFL as one of the measurement indicators to replace semiautomated methods.

Furthermore, when the ACCMetrics software is compared to the traditional method, there are negligible differences in the results. In Table 1, it can be seen that the ACCMetrics software recorded higher CNFD values, lower CNBD values, and lower CNFL values. Conversely, the CNFD has a weak correlation (ICC = 0.460, *p* < 0.001, r = 0.471, *p* < 0.001) between the two approaches. Figure 2 presents the reasons behind some differences, e.g., some nerve trunks were misidentified and a certain number of nerve bands and fibers were missed, as indicated by the arrows and circles in the images. Consequently, we must acknowledge that the two methods differ, and we do not recommend CNFD as the primary parameter to be measured.

However, we recommend combining the CNFD, CNBD, and CNFL to support the diagnosis of DED with ocular pain, with the CNFL serving as the most critical parameter. Patients with ocular pain have fewer SNP, such as lower CNFD, CNBD, and CNFL values, regardless of whether the data are obtained via NeuronJ or ACCMetrics (17). From Table 4, it is apparent that when NeuronJ was used, CNFD was decreased by 11.32%, CNBD was decreased by 31.19%, and CNFL was decreased by 18.99%. Following the implementation of ACCMetrics, CNFD was decreased by 22.48%, CNBD was decreased by 22.39%, and CNFL was decreased by 18.28%. The ROC parameters (Figure 5) also demonstrate that the three indexes are all highly efficient diagnostic tools. In addition, we recommend the CNFL obtained via NeuronJ and ACCMetrics as the recommended parameter. DED patients with ocular pain exhibit distinctive pathological changes in the SNP, which can be detected using IVCM, and nerve quantification can be used to confirm the diagnosis.

It is widely accepted that manual and semiautomated methods have some advantages, such as the highest degree of consistency and efficacy. The observers can obtain additional pathological details on factors, such as inflammatory cells, nerve ganglion, neuroma, and nerve endpoint, which helps them make a more accurate diagnosis of the situation (19). However, these methods are not only time and energy consuming, but also challenging to apply in a medical environment, particularly, when analyzing large amounts of data (18).

A fully automated method is well suited to clinical applications because it is operated automatically, allowing clinicians to conserve time and energy. A significantly large number of clinical samples can be assessed and the values of parameters can be calculated, facilitating a deeply comprehensive understanding of the symptoms, and consequently, highly effective treatment for the patients (20). Nevertheless, there are some disadvantages, such as the extremely high image quality requirements, such as image representativeness, clarity, and contrast. As indicated in Figure 2, the ACCMetrics software recognizes fewer nerve fibers and is not very capable of distinguishing nerve branches, inflammatory cells, nerve ganglions, neuromas, nerve endpoints, and other such entities in the cornea (16).

Simultaneously, our research does have some limitations. Standard IVCM generates narrow-field images of the cornea (400 × 400 μm) that each display an extremely small area (<1%). According to the study of Winter et al., averaging parameters from multiple IVCM images may not result in very well accurate predictions for the whole image region (21). Additionally, it requires manual selection of non-overlapping IVCM images from a larger pool of images acquired during the image acquisition phase. Meanwhile, Allgeier et al. developed a novel automated approach that incorporates directed eye movements to rapidly expand the captured SNP region and axial focus plane oscillations to ensure complete imaging of the SNP (22). Moreover, we learned from their work on CNFL that expanding the mosaic image area stabilizes the CNFL values and decreases the movement variation (23). Currently, there have been a number of reports using wide-field imaging tools to study SNP-related diseases, such as the study of severe diabetic foot deformity by Herlyn et al. (24), Andreasson et al.'s research on Parkinson's disease with restless legs syndrome (25), Sterenczak et al.'s research on atypical cellular elements of unknown origin of a diabetic cornea (26), Koschmieder et al.'s research on multiple myeloma (27), and so on. A significant advantage of the wide-field technique is its capacity to offer imaging data with a wider field of view, possibly allowing the physician to analyze identical tissue sections repeatedly (22). Moreover, it prevents subjective field selection, resulting in an objective perspective of the whole SNP architecture, enabling accurate analysis of SNP patterns and precise quantification of SNP parameters (28). However, the wide-field technique for mosaic images is generally more time-consuming and difficult to implement in terms of software and hardware (e.g., three connected PCs) configuration. In our study, due to a lack of appropriate software and hardware, we relied on the widely used standard IVCM image extraction approach (29). Our study applied the well-accepted method of averaging the values of the five sites to represent the overall SNP of the eye (11). In the process of photographing and image selection, we tried to select images containing non-repeating SNP. The study of Ahmad Kheirkhah supported that there are no significant variations in the mean sub-basal nerve and dendritic cell densities between the average values of three representative standard IVCM images and wide-field mosaic composite images (29). However, given that the images we analyzed were of cases of decreased nerve density, the wide-field composite images may more accurately represent the entire cornea. We anticipate further studies on the application of wide-field techniques on DED with ocular pain.

Our expectations and recommendations for the future of the ACCMetrics software are as follows: we anticipate that the structure will improve software detection while simultaneously increasing recognition in images with low contrast and poor clarity; improved detection of nerve bands, weak nerve fibers, inflammatory cells, nerve ganglions, neuromas, nerve endpoints, and other abnormalities in the nervous system, and detection of other irregularities in the corneal stroma.

In summary, the fully automated software ACCMetrics can be used to partly replace semiautomated method NeuronJ. For instance, a relatively precise measurement of the CNFL can denote a significant degree of corneal nerve loss in patients, which can be used as a reference for the diagnosis of DED with ocular pain. Present status demonstrates the clinical importance in various fields of medicine and not only in DED with ocular pain. It may be turned out as an early biomarker for many neurodegenerative diseases, such as diabetic peripheral neuritis, Parkinson's disease, and so on. Nevertheless, current software often incorrectly recognizes and underestimates nerve fibers and is unable to identify biological details in images (e.g., inflammatory cells). Therefore, we anticipate that the ACCMetrics software will be upgraded and further developed to meet the demands of clinical practice.

## Data Availability Statement

The original contributions presented in the study are included in the article/supplementary material, further inquiries can be directed to the corresponding author.

## Ethics Statement

The studies involving human participants were reviewed and approved by the Ethics Committee of The Second Affiliated Hospital at Zhejiang University School of Medicine. The patients/participants provided their written informed consent to participate in this study.

## Author Contributions

YZ wrote the manuscript. XH designed the research. YW and XH collected the data. YZ, YW, and WL analyzed the data. All authors contributed to the article and approved the submitted version.

## Funding

This work was supported by the National Natural Science Foundation of China (Grant Numbers: 81870624 and 82171013) and Major Science and Technology Projects of Zhejiang Province (Grant Number: 2022C03173).

## Conflict of Interest

The authors declare that the research was conducted in the absence of any commercial or financial relationships that could be construed as a potential conflict of interest.

## Publisher's Note

All claims expressed in this article are solely those of the authors and do not necessarily represent those of their affiliated organizations, or those of the publisher, the editors and the reviewers. Any product that may be evaluated in this article, or claim that may be made by its manufacturer, is not guaranteed or endorsed by the publisher.

## References

[1] Farrand K Fridman M Stillman I Schaumberg D. Prevalence of diagnosed dry eye disease in the United States among adults aged 18 years and older. Am J Ophthalmol. (2017) 182:90–8. 10.1016/j.ajo.2017.06.033 28705660

[2] Rouen P White M. Dry eye disease: prevalence, assessment, and management. Home Healthc Now. (2018) 36:74–83. 10.1097/NHH.0000000000000652 29498987

[3] Song P Xia W Wang M Chang X Wang J Jin S. Variations of dry eye disease prevalence by age, sex and geographic characteristics in China: a systematic review and meta-analysis. J Global Health. (2018) 8:020503. 10.7189/jogh.08.020503 30206477 PMC6122008

[4] Craig J Nichols K Akpek E Caffery B Dua H Joo C. TFOS DEWS II definition and classification report. Ocul Surf. (2017) 15:276–83. 10.1016/j.jtos.2017.05.008 28736335

[5] Belmonte C Nichols J Cox S Brock J Begley C Bereiter D. TFOS DEWS II pain and sensation report. Ocul Surf. (2017) 15:404–37. 10.1016/j.jtos.2017.05.002 28736339 PMC5706540

[6] Giannaccare G Pellegrini M Sebastiani S Moscardelli F Versura P Campos EC. In vivo confocal microscopy morphometric analysis of corneal subbasal nerve plexus in dry eye disease using newly developed fully automated system. Graefe's archive for clinical and experimental. Ophthalmology. (2019) 257:583–9. 10.1007/s00417-018-04225-7 30637452

[7] Patel S Hwang J Mehra D Galor A. Corneal Nerve Abnormalities in Ocular and Systemic Diseases. Exp Eye Res. (2021) 202:108284. 10.1016/j.exer.2020.108284 33045221

[8] Oliveira-Soto L Efron N. Morphology of corneal nerves using confocal microscopy. Cornea. (2001) 20:374–84. 10.1097/00003226-200105000-00008 11333324

[9] Shetty R Sethu S Deshmukh R Deshpande K Ghosh A Agrawal A. Corneal dendritic cell density is associated with subbasal nerve plexus features, ocular surface disease index, and serum vitamin d in evaporative dry eye disease. Biomed Res Int. (2016) 2016:4369750. 10.1155/2016/4369750 26904676 PMC4745572

[10] Yavuz-Saricay L Bayraktutar B Kenyon B Hamrah P. Concurrent ocular pain in patients with neurotrophic keratopathy. Ocul Surf. (2021). 10.1016/j.jtos.2021.08.003 34411735 PMC8560561

[11] Chin J Yang L Ji A Nubile M Mastropasqua L Allen J. Validation of the use of automated and manual quantitative analysis of corneal nerve plexus following refractive surgery. Diagnostics (Basel, Switzerland). (2020) 10. 10.3390/diagnostics10070493 32708510 PMC7400400

[12] Tavakoli M Ferdousi M Petropoulos IN Morris J Pritchard N Zhivov A. Normative values for corneal nerve morphology assessed using corneal confocal microscopy: a multinational normative data set. Diabetes Care. (2015) 38:838–43. 10.2337/dc14-2311 25633665 PMC4407754

[13] Henley R Chandrasekaran V Giulivi C. Computing neurite outgrowth and arborization in superior cervical ganglion neurons. Brain Res Bullet. (2019) 144:194–9. 10.1016/j.brainresbull.2018.12.001 30529562 PMC6994235

[14] Liu Y Lin M Mehta J. Analysis of corneal nerve plexus in corneal confocal microscopy images. Neural Regen Res. (2021) 16:690–1. 10.4103/1673-5374.289435 33063728 PMC8067927

[15] Qazi Y Hurwitz S Khan S Jurkunas U Dana R Hamrah P. Validity and reliability of a novel ocular pain assessment survey (OPAS) in quantifying and monitoring corneal and ocular surface pain. Ophthalmology. (2016) 123:1458–68. 10.1016/j.ophtha.2016.03.006 27089999 PMC5512896

[16] Dehghani C Pritchard N Edwards K Russell AW Malik RA Efron N. Fully automated, semiautomated, and manual morphometric analysis of corneal subbasal nerve plexus in individuals with and without diabetes. Cornea. (2014) 33:696–702. 10.1097/ICO.0000000000000152 24886994

[17] Kheirkhah A Dohlman TH Amparo F Arnoldner MA Jamali A Hamrah P. Effects of corneal nerve density on the response to treatment in dry eye disease. Ophthalmology. (2015) 122:662–8. 10.1016/j.ophtha.2014.11.006 25542519 PMC4372494

[18] Wu PY Wu JH Hsieh YT Chen LC Cheng T Wu PY. Comparing the results of manual and automated quantitative corneal neuroanalysing modules for beginners. Sci Rep. (2021) 11:18208. 10.1038/s41598-021-97567-y 34521890 PMC8440557

[19] Wu LQ Mou P Chen ZY Cheng JW Le QH Cai JP. Altered corneal nerves in Chinese thyroid-associated ophthalmopathy patients observed by in vivo confocal microscopy. Med Sci Monit. (2019) 25:1024–31. 10.12659/MSM.912310 30724266 PMC6373222

[20] Recchioni A Sisó-Fuertes I Hartwig A Hamid A Shortt AJ Morris R. Short-term impact of FS-LASIK and SMILE on dry eye metrics and corneal nerve morphology. Cornea. (2020) 39:851–7. 10.1097/ICO.0000000000002312 32243424

[21] Winter K Scheibe P Köhler B Allgeier S Guthoff RF Stachs O. Local variability of parameters for characterization of the corneal subbasal nerve plexus. Curr Eye Res. (2016) 41:186–98. 10.3109/02713683.2015.1010686 25803579

[22] Allgeier S Bartschat A Bohn S Peschel S Reichert KM Sperlich K. 3D confocal laser-scanning microscopy for large-area imaging of the corneal subbasal nerve plexus. Sci Rep. (2018) 8:7468. 10.1038/s41598-018-25915-6 29749384 PMC5945773

[23] Allgeier S Winter K Bretthauer G Guthoff RF Peschel S Reichert KM. A novel approach to analyze the progression of measured corneal sub-basal nerve fiber length in continuously expanding mosaic images. Curr Eye Res. (2017) 42:549–56. 10.1080/02713683.2016.1221977 27767360

[24] Herlyn A Prakasam RK Peschel S Allgeier S Kohler B Winter K. Corneal subbasal nerve plexus changes in severe diabetic charcot foot deformity: a pilot study in search for a DNOAP biomarker. J Diabetes Res. (2018) 2018:5910639. 10.1155/2018/5910639 30525053 PMC6247393

[25] Andréasson M Lagali N Badian RA Utheim TP Scarpa F Colonna A. Parkinson's disease with restless legs syndrome-an in vivo corneal confocal microscopy study. NPJ Parkinson's Disease. (2021) 7:4. 10.1038/s41531-020-00148-5 33402694 PMC7785738

[26] Sterenczak KA Stachs O Marfurt C Matuszewska-Iwanicka A Stratmann B Sperlich K. Atypical cellular elements of unknown origin in the subbasal nerve plexus of a diabetic cornea diagnosed by large-area confocal laser scanning microscopy. Diagnostics (Basel). (2021) 11:154. 10.3390/diagnostics11020154 33494468 PMC7911241

[27] Koschmieder A Stachs O Kragl B Stahnke T Sterenczak KA Henze L. Non-invasive detection of corneal sub-basal nerve plexus changes in multiple myeloma patients by confocal laser scanning microscopy. Biosci Rep. (2020) 40:BSR20193563. 10.1042/BSR20193563 33026069 PMC7578619

[28] Badian RA Allgeier S Scarpa F Andréasson M Bartschat A Mikut R. Wide-field mosaics of the corneal subbasal nerve plexus in Parkinson's disease using in vivo confocal microscopy. Sci Data. (2021) 8:306. 10.1038/s41597-021-01087-3 34836991 PMC8626466

[29] Kheirkhah A Muller R Mikolajczak J Ren A Kadas EM Zimmermann H. Comparison of standard versus wide-field composite images of the corneal subbasal layer by in vivo confocal microscopy. Investig Ophthalmol Visual Sci. (2015) 56:5801–7. 10.1167/iovs.15-17434 26325419 PMC4559214

